# Diagnostic utility of *LunX *mRNA in peripheral blood and pleural fluid in patients with primary non-small cell lung cancer

**DOI:** 10.1186/1471-2407-8-156

**Published:** 2008-05-31

**Authors:** Min Cheng, Yongyan Chen, Xiaoqing Yu, Zhigang Tian, Haiming Wei

**Affiliations:** 1 Hefei National Laboratory for Physical Sciences at Microscale and School of Life Sciences, University of Science & Technology of China, Hefei, Anhui 230027, PR China; 2 The First People's Hospital of Hefei, Hefei, Anhui 230031, PR China

## Abstract

**Background:**

Progress in lung cancer is hampered by the lack of clinically useful diagnostic markers. The goal of this study was to provide a detailed evaluation of lung cancer tumor markers indicative of molecular abnormalities and to assess their diagnostic utility in non-small cell lung cancer (NSCLC) patients.

**Methods:**

Quantitative real-time RT-PCR was used to determine *LunX, CK19, CEA, VEGF-C *and *hnRNP A2/B1 *mRNA levels in peripheral blood and pleural fluid from NSCLC patients, compared with those from patients with other epithelial cancer (esophagus cancer and breast cancer), benign lung disease (pneumonia and tuberculo pleurisy) and from healthy volunteers.

**Results:**

In peripheral blood *LunX *mRNA was detectable in 75.0% (33/44) of patients with NSCLC, but not in patients with other epithelial cancer (0/28), benign lung disease (0/10) or in healthy volunteers (0/15). In contrast, all other genetic markers were detected in patients with either NSCLC, other epithelia cancer or benign lung disease, and in healthy volunteers. The expression level and positive rate of *LunX *mRNA in peripheral blood correlated with the pathologic stage of NSCLC (P < 0.001 and P = 0.010 respectively). Furthermore, *LunX *mRNA was detected in 92.9% (13/14) of malignant pleural fluid samples and was the only marker whose expression level was significantly different between malignant and benign pleural fluid (P < 0.001). Additionally, expression of *LunX *mRNA in the peripheral blood of NSCLC patients decreased shortly after clinical treatment (P = 0.005).

**Conclusion:**

Of several commonly used genetic markers, *LunX *mRNA is the most specific gene marker for lung cancer and has potential diagnostic utility when measured in the peripheral blood and pleural fluid of NSCLC patients.

## Background

Lung cancer is one of the leading causes of cancer death and has become an increasingly urgent worldwide health problem. Progress in lung cancer treatment is hampered by a lack of diagnostic markers useful in clinical practice. Tumor markers, including carcinoembryonic antigen (CEA), neuron-specific enolase (NSE), squamous cell carcinoma (SCC) antigen, cytokeratin 19 (CK19), vascular endothelial growth factor-C (VEGF-C), heterogeneous ribonuclear proteins A2/B1 (hnRNP A2/B1), muc1, BJ-TSA-9, KS1/4 and lung-specific X protein (LunX), have been investigated for their putative diagnostic and prognostic value for lung cancer [1-7]. On the basis of RT-PCR analysis, *CEA *mRNA in blood cells or in lymph nodes and *CK19 *mRNA in mediastinal lymph nodes have been suggested as promising tools for the detection of micrometastatic cells in patients with lung cancer [8-11]. *KS1/4 *was shown to be the most sensitive marker for detecting metastatic NSCLC in mediastinal lymph nodes using real-time RT-PCR [6]. Determining CEA and NSE in pleural fluid could enhance the diagnostic yield for malignant effusion associated with lung cancer [12]. Additionally, accumulating evidence suggests that hnRNP B1 expression may be useful for the early diagnosis of lung cancer, provided that expression levels can be accurately quantified [5]. *LunX*, a novel human lung-specific gene, has been reported to be a superior diagnostic marker for the detection of micrometastases in lymph nodes and peripheral blood of NSCLC patients [1,6,13]. Although lots of studies have provided suggestive results, a definitive assessment of the relative value of these molecular markers for lung cancer is lacking.

A demonstration of the diagnostic utility of these various tumor markers requires a detailed, direct comparison using reliable, sensitive methodologies. Quantitative real-time RT-PCR is a development of the RT-PCR procedure, which is simple, rapid and automated. Most importantly, real-time RT-PCR analysis can yield accurate estimates of gene expression levels, differentiating between baseline levels of gene expression in normal tissue and increased levels in cancer cells [14,15]. Molecular diagnosis using RT-PCR technique can detect tumor marker-expressing cells undetectable by other means in patients with localized or metastatic cancer, and may offer the most effective solution for detecting micrometastases at the molecular level in various types of cancer patients [16].

The purpose of this study was to evaluate the known molecular markers,*LunX, CK19, CEA, VEGF-C *and *hnRNP A2/B1*, for their expression in lung cancer cells in peripheral blood and pleural fluid using real-time RT-PCR, with the ultimate goal of establishing a more reliable molecular diagnostic method as an adjunct to clinical decision-making.

## Methods

### Patients and clinical procedures

Patients with pathologically proven non-small cell lung cancer (NSCLC) identified by routine imaging and cytologic assessments were eligible for the study. The clinical characteristics of patients (peripheral blood and pleural fluid groups) were shown in Table 1. Patients with other epithelial cancer, including esophagus and breast cancer, were studied as a control. Additionally, 12 patients with NSCLC were investigated before and after treatment (Table 2). Patients with a history of malignancy were excluded from the study. Informed consent was obtained from each subject and the research was performed in compliance with the principles enunciated in the Helsinki Declaration with the approval of the Ethics Committee of the University of Science and Technology of China.

**Table 1:** Clinical characteristics of patients in this study

	**Peripheral blood**					**Pleural fluid**	
		
	**Lung cancer**	**Esophagus cancer**	**Breast cancer**	**Pneumonia**	**Healthy**	**Lung cancer**	**Tuberculo pleurisy**
**Age mean (min-max)**	67(36–89)	62(39–85)	51(35–82)	67(52–83)	42(25–60)	66(36–85)	64(24–85)
**Sex (M/F)**	31/13	5/3	0/20	8/2	8/7	9/5	12/2
**Pathologic stage**							
**I**	8	1	3	-	-	-	-
**II**	8	3	8	-	-	-	-
**III**	14	2	5	-	-	5	-
**IV**	14	2	4	-	-	9	-
**Pathologic Typing**							
	Squ 26	Squ 6	-	-	-	Squ 9	-
	Ade 16	Ade 2	-	-	-	Ade 5	-
	LCLC 2	-	-	-	-	LCLC	-
**Total number**	44	8	20	10	15	14	14

Pathologic stage was determined as I, II, III or IV according to the American Joint Committee On Cancer (AJCC) TNM system. Squ = squamous epithelial carcinoma; Ade = adenocarcinoma; LCLC = large cell lung cancer.

**Table 2:** Clinical characteristics and treatments of 12 NSCLC patients

**Patient**	**Sex**	**Age (y)**	**Pathologic Typing**	**Pathologic Stage**	**Means of treatment**
**1**	F	64	LCLC	II	chemo-therapy for 7 days
**2**	M	70	Squ	III	chemo-therapy for 7 days
**3**	M	70	Squ	III	chemo-therapy for 7 days
**4**	F	36	Squ	III	chemo-therapy for 7 days
**5**	M	75	Ad	IV	chemo-therapy for 7 days
**6**	M	62	Squ	IV	interventional therapy
**7**	M	65	Ad	II	operation
**8**	F	58	Squ	IV	chemo-therapy for 7 days
**9**	M	55	Squ	III	interventional therapy
**10**	M	58	Squ	II	operation
**11**	M	68	Ad	I	operation
**12**	M	61	Ad	II	operation

Peripheral blood samples from each patient were collected 1 day before and 7 days after treatment. Pathologic stage was determined as I, II, III or IV according to the American Joint Committee On Cancer (AJCC) TNM system. Squ = squamous epithelial carcinoma; Ade = adenocarcinoma; LCLC = large cell lung cancer.

### Collection of specimens

3 ml peripheral blood (the first 2 ml peripheral blood had been discard for detection convenience) was collected and treated with RBC lysis buffer (RX-2-1-2, U-gene, China), and then nucleated cells were collected for the detection of biomarker mRNA. 10 ml pleural fluid was inspired from indicated patients, and centrifugated at 3500 rpm for 10 min to pellet cells.

### RNA extraction and cDNA synthesis

All reagents were purchased from Invitrogen. Total cellular RNA was extracted using the TRIZOL reagent according to the protocol provided by the manufacturer. cDNA was generated form total RNA by reverse transcription (RT) in a reaction containing 4 μg total RNA, 5 μM oligo dT, 0.5 mM dNTP, 8 μl 5×Buffer, 10 mM DTT, 56 units RNase inhibitor, 400 units of M-MLV and distilled water (ultrapure, DNase and RNase free) in a total volume of 40 μl. The RT reaction was performed at 37°C for 50 minutes, followed by heating at 70°C for 15 minutes. All of the steps were performed using sterile technique in areas designated for RNA extraction and RT-PCR.

### Real-time PCR

Quantitative real-time PCR was performed using real-time Taq-Man technology and an ABI PRISM 7000 sequence detector (Applied Biosystems, Foster City, CA). Gene-specific primers and Taq-Man probes were shown in Table 3. The standard reaction contained 25 μl 2×PCR buffer (ABsolute™ QPCR Mix, AB-1140/b), 0.5 U uracil N-glycosylase (UNG) Erase enzyme (Invitrogen, Cat NO.18054-015), 5 μl cDNA template, 0.4 μM forward and reverse primers, 0.25 μM hybridization probe in a total volume of 50 μl. The initial PCR step was at 37°C for 10 min to activate UNG erase, followed by a 15 min hold at 95°C. PCR reactions were performed using a total of 50 cycles consisting of a 15 s melt at 95°C, followed by a 1 min annealing/extension at 60°C for *CK19, CEA, VEGF-C, hnRNP A2/B1 *and *β-actin *or a 1 min annealing/extension at 56°C for *LunX*. During the DNA polymerization, the Taq-Man probe was hydrolyzed and fluorescence emitted. When the fluorescence signal reached 10 SDs of background, the threshold cycle (Ct) was noted. Each sample was analyzed in triplicate for each target gene, and mRNA was quantified by the standard curve method.

**Table 3:** Primer pair and probe of each biomarker for real-time PCR

**Gene**	**Sequence of selected primer pair (5'-3')**	**Probe **(5'-FAM---TAMRA-3')	**Amplicon length (bp)**	**Ref**
***LunX***	GCCTCATTGTCTTCTACGGGCTGTT; CTGAGGGCATTTGTCAAGCTTCCT	CAGACCATGGCCCAGTTTGGAGGCCTG	156	This work
***CK19***	ACTACAGCCACTACTACACGAC; CAGAGCCTGTTCCGTCTCAAAC	TCTGGCTGCAGATGACTTCCGAACCA	149	[17]
***CEA***	AATAACGGGACCTATGCCTGTTT; CCCCAACCAGCACTCCAAT	CATCTGGAACTTCTCCTGGTCTCTCAGCTG	151	This work
***VEGF-C***	TTCATTCCATTATTAGACGTTCCCT; GATTATTCCACATGTAATTGGTGGG	CCAGCAACACTACCA CAGTGTCAGGCA	94	[18]
***hnRNP A2/B1***	GACTGTGTGGTAATGAGGGATCCT; GCTCAACTACTCTCCCATCAATTGA	CATGGCTGAGGTTGATGCTGCCAT	133	This work
***β-actin***	TTGCCGACAGGATGCAGAA; GCCGATCCACACGGAGTACTT	TCATTGCTCCTCCTGAGC	101	This work

Forward primer was shown as upper sequence and reverse primer was the lower sequence in the respective primer pair. The primers and probes for LunX (NM_130852), CEA (NM_004363), hnRNP A2/B1 (NM_031243) and β-actin (NM_001101.2) were designed using the Primer Express Software (Applied Biosystems). All primers were desalted when purified and the probes were HPLC purified.

### Preparation of standard curves

*LunX, CK19, CEA, VEGF-C, hnRNP A2/B1 *and *β-actin *cDNA were generated from A549, SK-BR-3, SK-BR-3, K562, A549 and A549 cells (American Type Culture Collection, Rockville, MD), respectively, using the specific primers in Table 3. The PCR reaction consisted of an initial denaturation step at 94°C for 3 min, followed by 35 cycles of denaturation at 94°C, annealing at 60°C for *CK19, CEA, VEGF-C, hnRNP A2/B1 *and *β-actin *or at 56°C for *LunX*, and extension at 72°C. After purification using a gel band purification kit (Invitrogen), the amplicon was ligated into a TA-cloning vector pCR 2.1 (Invitrogen, Groningen, The Netherlands) and then used to transform competent E. coli (DH5α). The sequence of the insert was verified using an ABI Prism BigDye terminator cycle sequencing ready reaction kit (PE Biosystems, Foster City, CA) and an ABI Prism 377 DNA sequencer. The number of plasmid copies was determined according to the formula: Copy number = $\frac{mass(g)}{molecule-weight(g/mol)}\times 6.02\times 10^{23}mol^{-1}$. Serial dilutions from 1 × 10^2 ^to 1 × 10^8 ^copies of plasmids per microliter were made in TE buffer (10 mM Tris, 0.1 M EDTA, pH 8.0) in tubes lubricated with silicon and plasmids were detected by real-time PCR as described above. Standard curves were determined by plotting the Ct value against an initial copy number of standards (serially diluted plasmids) for *LunX, CK19, CEA, VEGF-C*, *hnRNP A2/B1 and β-actin *mRNA. Copy numbers of each gene marker of clinical samples were calculated by interpolating sample Ct value with standard curves of Ct values generated by serial dilution of the corresponding standard. The copy numbers of *LunX, CK19, CEA, VEGF-C *and *hnRNP A2/B1*were all further normalized to the copy number of *β-actin *in each sample.

### Statistical analysis

SDS 1.0 software was used to analyze the results of real-time PCR. A regression analysis was applied to standard curves. K Independent Samples Test (Media Test) was used to compare the gene expression levels in peripheral blood among NSCLC patients at different pathologic stages. Mann-Whitney U Test was used to compare the gene expression levels in pleural fluid between NSCLC and tuberculo pleurisy patients. Wilcoxon Signed Ranks Test was used for the analysis of gene expression levels in peripheral blood of NSCLC patients before and after clinical treatment. In cases where the results of gene expression were negative, the data were treated as 0 for statistical convenience. χ^2 ^test was used to analyze the positive detection rate. A value of P < 0.05 (2-tailed test) was considered significant.

## Results

### Establishment of real-time RT-PCR procedures for the gene markers

**Figure 1:**
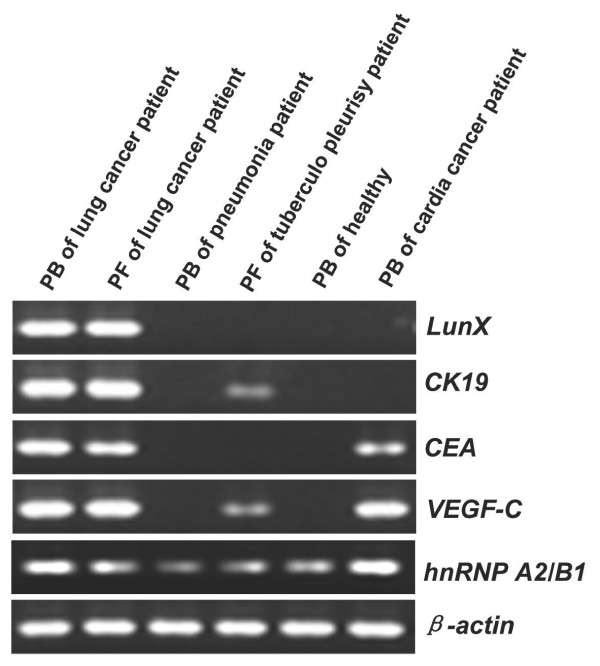
**Products of each gene marker by real-time RT-PCR**. Each biomarker was detected by real-time RT-PCR in the indicated samples, as described in methods. Products were separated by electrophoresis and visualized in ethidium bromide-stained agarose gels. PB = Peripheral blood; PF = Pleural fluid.

The standard curves for *LunX*, *CK19*, *CEA*, *VEGF-C*, *hnRNP A2/B1 *and *β-actin *mRNA detection were established using specific plasmids, as described in methods. The amplification efficiencies of the PCR reactions were 99.3% for *hnRNPA2/B1*, 100% for *LunX*, *CEA *and *VEGF-C*, 101% for *β-actin *and 103% for *CK19*, indicating a near-perfect doubling (100%) of product with each amplification cycle. Using established procedures, each biomarker was detected in specific specimens and the products were visualized on ethidium bromide-stained agarose gel (Figure 1). The expression patterns were consistent with the performance of each biomarker in these specimens, which further validated the RT-PCR procedures established for each biomarker in this study.

### *LunX *mRNA is the most specific gene marker for NSCLC cells in peripheral blood

**Figure 2:**
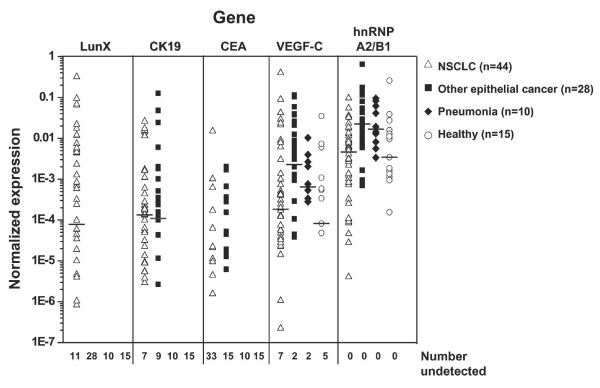
***LunX *mRNA is the most specific gene marker for lung cancer cells in peripheral blood**. *LunX, CK19, CEA, VEGF-C, hnRNP A2/B1 *and *β-actin *mRNA from each peripheral blood sample were detected by real-time RT-PCR, and mRNA copy number was determined by reference to the standard curve, as described in methods. The copy number of each mRNA was further normalized as the ratio to the copy number of *β-actin *in all samples. For each gene marker, when the copy number was less than 100, it could not be detectable as a negative case. All the negative results of each indicated gene were shown as number undetected. The median is marked as "**---**" in each group. Where the frequency of negative cases is > 50%, the median cannot be shown.

As shown in Figure 2, *LunX *mRNA was detectable in the peripheral blood from only NSCLC patients; *LunX *mRNA was not detectable in the peripheral blood from patients with other epithelial cancer, patients with pneumonia, or healthy volunteers. In contrast, *CK19 *mRNA could be detectable not only in peripheral blood from NSCLC patients but also in blood from other epithelial cancer patients, and there was no significant difference in the expression levels of *CK19 *mRNA between the two groups (P = 0.667). As with CK19, the difference in *CEA *mRNA between the two groups was not significant (P = 0.050). *VEGF-C *and *hnRNP A2/B1*mRNA were present at high levels in peripheral blood samples from NSCLC patients, other epithelial cancer patients, pneumonia patients and the healthy, obviously ruling out these genes as effective gene markers for lung cancer cells in peripheral blood.

Further, for NSCLC, the positive detection rate of *LunX *mRNA was 75.0% (33 of 44) in peripheral blood, which was similar to the positive detection rate of *CK19 *mRNA (84.1%, 37 of 44) (P = 0.290), but much higher than the positive detection rate of *CEA *mRNA (25.0%, 11 of 44) (P < 0.001) (Table 4). Although *CK19 *mRNA appeared to be a sensitive NSCLC detector in peripheral blood, there was no significant difference in the positive detection rate between NSCLC and other epithelial cancer groups (P = 0.106). The frequency of *CEA *mRNA detection in peripheral blood was likewise not significantly different between NSCLC and other epithelial cancer groups (P = 0.060), providing further confirmation that *CK19 *and *CEA *mRNA were not specific to lung cancer cells.

**Table 4:** Number of positive cases by each biomarker in peripheral blood

**Group**	**Total number**	**Positive Detection Rate**				
		
		***LunX***	***CK19***	***CEA***	***VEGF-C***	***hnRNP A2/B1***
**NSCLC**	44	33/44 (75.0%)	37/44 (84.1%)	11/44 (25.0%)	37/44 (84.1%)	44/44 (100%)
**Other epithelial cancer**	28	0/28 (0%)	19/28 (67.9%)	13/28 (46.4%)	26/28 (92.9%)	28/28 (100%)
**Pneumonia**	10	0/10 (0%)	0/10 (0%)	0/10 (0%)	8/10 (80.0%)	10/10 (100%)
**Healthy **	15	0/15 (0%)	0/15 (0%)	0/15 (0%)	10/15 (66.7%)	15/15 (100%)

***P value***		< 0.001*	< 0.001*	0.002*	0.174*	-
		< 0.001^#^	0.106^#^	0.060^#^	-	-
		/	0.290^$^	< 0.001^$^	-	-

For each gene marker, when the copy number was less than 100, it could not be detectable as a negative case using our established real-time PCR assay. The positive detection rate was calculated by the number of positive cases/total number. * represented the analysis among NSCLC, other epithelial cancer, pneumonia and healthy groups for each biomarker. # represented the analysis between NSCLC and other epithelial cancer groups for each biomarker. $ represented the analysis between the biomarker and LunX for NSCLC. P values were calculated using χ^2 ^test.

### Expression of *LunX *mRNA in peripheral blood is correlated with the pathologic stage of NSCLC

**Figure 3:**
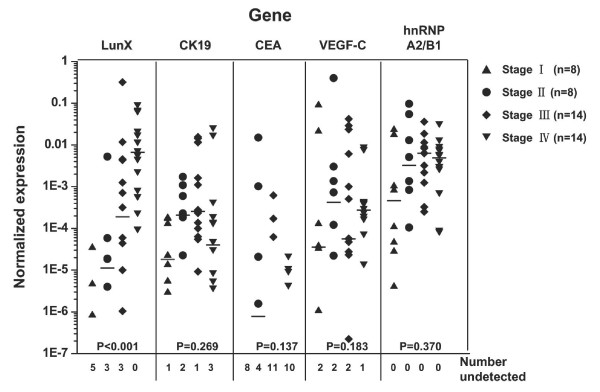
**Expression level of *LunX *mRNA in peripheral blood is correlated with the pathologic stage of NSCLC**. *LunX, CK19, CEA, VEGF-C, hnRNP A2/B1 *and *β-actin *mRNA from each peripheral blood sample were detected by real-time RT-PCR, and mRNA copy number was determined by reference to the standard curve, as described in methods. The copy number of each mRNA was further normalized as the ratio to the copy number of *β-actin*. For each gene marker, when the copy number was less than 100, it could not be detectable as a negative case. All the negative results of each indicated gene were shown as number undetected. The median is marked as "**---**" in each group. Where the frequency of negative cases is > 50%, the median cannot be shown. The K Independent Samples Test (Media Test) was used to analyze gene expression levels among NSCLC patients at different pathologic stages. P < 0.05 (2-tailed test) was considered significant.

The correlation of *LunX *mRNA expression in peripheral blood of NSCLC patients and clinical factors was further investigated, as shown in Table 5. Gender, age and pathologic type were not associated with the positive detection rate of *LunX *mRNA in peripheral blood (P = 0.461, 0.425 and 0.482 respectively). However, there was an association between different pathologic stages and the *LunX *mRNA positive detection rate in peripheral blood (P = 0.010), which increased with increasing clinical severity. A similar relationship between the levels of *LunX *mRNA expression in the peripheral blood of NSCLC patients and pathological stages was found (P < 0.001) (Figure 3). There was no significant difference in the mRNA expression levels of the other gene markers, *CK19 *(P = 0.269), *CEA *(P = 0.137), *VEGF-C *(P = 0.183) or *hnRNP A2/B1 *(P = 0.370), in peripheral blood among NSCLC patients at different pathologic stages (Figure 3).

**Table 5:** Correlation between *LunX *mRNA in the peripheral blood and clinical factor in NSCLC patients

**Clinical factor**	**Positive cases/total cases**
**Sex**	
*P value*	0.461
Male	22/31 (71.0%)
Female	11/13 (84.6%)
**Age**	
*P value*	0.425
≤ 60	7/11 (63.6%)
> 60	26/33 (78.8%)
**Pathologic Type **	
*P value*	0.482
Squ	18/26 (69.2%)
Ade	13/16 (81.3%)
LCLC	2/2 (100%)
**Pathologic Stage**	
*P value*	0.010
I	3/8 (37.5%)
II	5/8 (62.5%)
III	11/14 (78.6%)
IV	14/14 (100%)

P values were calculated using χ^2 ^test.

### *LunX *mRNA is the most specific and sensitive gene marker for NSCLC cells in pleural fluid

**Figure 4:**
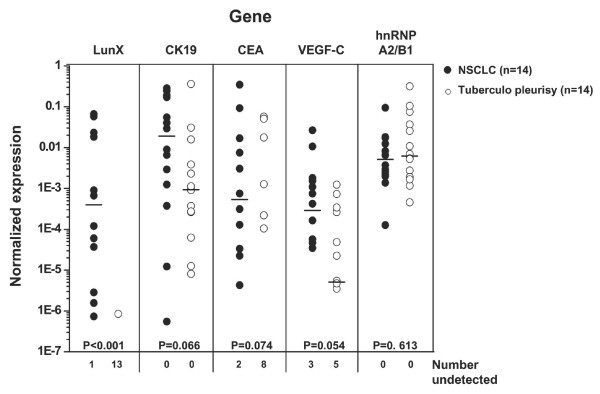
***LunX *mRNA is the most specific gene marker with high sensitivity for NSCLC cells in pleural fluid**. *LunX, CK19, CEA, VEGF-C, hnRNP A2/B1 *and *β-actin *mRNA from each pleural fluid sample were detected by real-time RT-PCR, and mRNA copy number was determined by reference to the standard curve, as described in methods. The copy number of each mRNA was further normalized as the ratio to the copy number of *β-actin*. For each gene marker, when the copy number was less than 100, it could not be detectable as a negative case. All the negative results of each indicated gene were shown as number undetected. The median is marked as "**---**" in each group. Where the frequency of negative cases is > 50%, the median cannot be shown. The Mann-Whitney U Test was used to compare the gene expression levels between NSCLC and tuberculo pleurisy groups. P < 0.05 (2-tailed test) was considered significant.

Pleural fluid can be caused by several kinds of diseases, including malignant lung cancer and benign lung disease. Distinguishing between malignant pleural fluid and benign pleural fluid is an urgent clinical concern. As shown in Figure 4 and Table 6, the detection rates of *LunX *(92.9%), *CK19 *(100%), *CEA *(85.7%), *VEGF-C *(78.6%) and *hnRNP A2/B1 *(100%) in the malignant pleural fluid from NSCLC patients were all high. However, only *LunX *mRNA was appropriately infrequent in the benign pleural fluid from patients with tuberculo pleurisy (7.1%, 1 of 14); all remaining markers were present in the benign pleural fluid at high frequencies (100%, 42.9%, 64.3% and 100% for *CK19, CEA, VEGF-C *and *hnRNP A2/B1 *mRNA, respectively) (Figure 4, Table 6). There were also no significant differences in the expression levels of *CK19 *(P = 0.066), *CEA *(P = 0.074),*VEGF-C *(P = 0.054), or *hnRNP A2/B1 *(P = 0.613) mRNA between malignant and benign pleural fluid (Figure 4). Only the expression level of *LunX *mRNA was significantly different between malignant and benign pleural fluid (P < 0.001) (Figure 4). Thus, *LunX *mRNA was the most specific biomarker with high sensitivity (13/14, 92.9%) among these detected markers for the differential diagnosis of NSCLC from pleural fluid.

**Table 6:** Number of positive cases by each biomarker in pleural fluid

**Group**	**Total number**	**Positive Detection Rate**				
		
		***LunX***	***CK19***	***CEA***	***VEGF-C***	***hnRNP A2/B1***
**NSCLC**	14	13/14 (92.9%)	14/14 (100%)	12/14 (85.7%)	11/14 (78.6%)	14/14 (100%)
**Tuberculo pleurisy**	14	1/14 (7.1%)	14/14 (100%)	6/14 (42.9%)	9/14 (64.3%)	14/14 (100%)

***P value***		< 0.001	-	0.018	0.678	-

For each gene marker, when the copy number was less than 100, it could not be detectable as a negative case using our established real-time PCR assay. The positive detection rate was calculated by the number of positive cases/total number. P values were calculated using χ^2 ^test.

### Expression of *LunX *mRNA in peripheral blood decreases shortly following treatment of NSCLC

**Figure 5:**
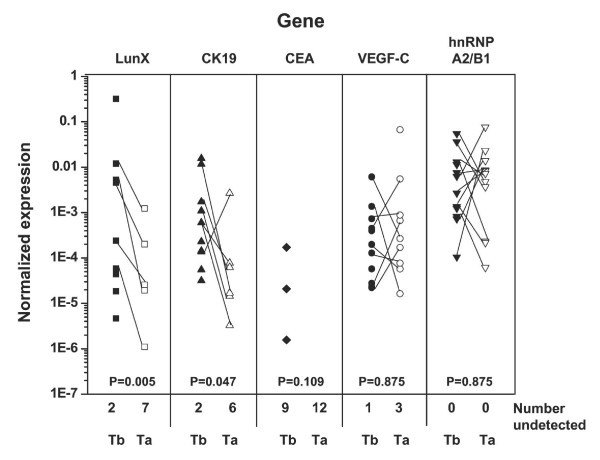
**Expression of *LunX *mRNA in peripheral blood decreases shortly following the treatment of NSCLC**. Peripheral blood samples from 12 NSCLC patients were collected 1 day before and 7 days after treatment as shown in Table 2. *LunX, CK19, CEA, VEGF-C, hnRNP A2/B1 *and *β-actin *mRNA were detected by real-time RT-PCR, and mRNA copy number was determined by reference to the standard curve, as described in methods. "Tb" represents 1 day before treatment and "Ta" represents 7 days after treatment. The copy number of each mRNA was further normalized as the ratio to the copy number of *β-actin*. For each gene marker, when the copy number was less than 100, it could not be detectable as a negative case. All the negative results of each indicated gene were shown as number undetected. The Wilcoxon Signed Ranks Test was used to analyze the gene expression levels before and after clinical treatment. P < 0.05 (2-tailed test) was considered significant.

An important attribute of a clinically useful diagnostic marker is the ability to promptly demonstrate the status of lung cancer after relevant treatments. In this study, *LunX, CK19, CEA, VEGF-C *and *hnRNP A2/B1 *mRNA in peripheral blood were tracked in 12 NSCLC patients before and after treatments (Table 2). As shown in Figure 5, the expression levels of *LunX and CK19 *mRNA in peripheral blood decreased significantly in NSCLC patients after treatment (P = 0.005 and P = 0.047, respectively). Treatment did not significantly affect the expression of *VEGF-C *or *hnRNP A2/B1 *mRNA (P = 0.875 and P = 0.875, respectively) (Figure 5), which, as demonstrated above, were detectable at high levels in peripheral blood from both patients and healthy (Figure 2). There was also no significant difference in the expression of *CEA *mRNA after treatment (P = 0.109) (Figure 5). These results, taken together with the demonstrated specificity of *LunX *mRNA for lung cancer cells and the correlation of *LunX *mRNA levels with NSCLC pathologic stages, indicated that *LunX *mRNA in peripheral blood might be a useful diagnostic marker for assessing the therapeutic effect on lung cancer.

## Discussion

The current staging evaluation for lung cancer is based on the presence or absence of disease in lymph nodes (hilar and mediastinal) and distant organs (principally bone, brain, adrenal glands, and liver) [19]. Approximately 35% of patients are diagnosed at early stage and, as such, are candidates for curative lung resection. However, 50% of these patients will develop metastases and die from their disease. Furthermore, about one fourth of patients at the earliest stage of NSCLC (pathologically confirmed stage I) die of tumor recurrence after radical surgery, indicating that undetected metastases are present at the time of surgery and demonstrating that conventional staging techniques lack the sensitivity necessary to properly characterize patients [20,21]. Cancer cells can be released from a primary site and spread *via *the bloodstream to form a micrometastatic deposit in distant organs [1]. However, due to their extremely low concentration, these circulating tumor cells in peripheral blood are difficult to detect. Thus, developing sensitive and specific detection methods for cancer cells in peripheral blood may have important diagnostic, prognostic and therapeutic implications [22].

In this study, we have developed quantitative real-time RT-PCR procedures to detect potentially diagnostic tumor markers (Figure 1). The detection of metastatic cancer cells by RT-PCR is possible because cancer cells continue to express genetic markers specific to the tissue from which they originate, but which are not normally expressed in tissue compartments that frequently harbor metastatic foci [23,24]. Using this RT-PCR approach, which is ideal for the detection of genes expressed at low levels [13], we have assessed the expression of the known molecular markers,*LunX, CK19, CEA, VEGF-C *and *hnRNP A2/B1*, in lung cancer cells in peripheral blood and pleural fluid. Although *LunX *has been previously reported to be the most sensitive marker among the five genes *LunX, muc1, KS1/4, CEA and CK19*, for detecting circulating NSCLC cells by real-time RT-PCR in a study distinguishing patients with NSCLC from healthy volunteers, the specificity of *LunX *for lung cancer cells has not been tested [13]. Ours is the first study to directly compare the expression of *LunX *with other biomarkers in peripheral blood and pleural fluid, not only from NSCLC patients but also from patients with other epithelial cancer or benign lung disease and healthy volunteers. We have found that *LunX *mRNA is the most specific marker for lung cancer cells in peripheral blood (Figure 2, Table 4) and pleural fluid (Figure 4, Table 6). Compared with *LunX *mRNA, *CK19 *and *CEA *mRNA were over-expressed in other epithelial cancers, such as breast and esophagus cancer (Figure 2, Table 4), thus limiting their specificity for detection of lung cancer cells in peripheral blood. Because of lack of specificity, *VEGF-C *and *hnRNP A2/B1*mRNA were found to be similarly ineffective as genetic markers for lung cancer cells in our quantitative real-time RT-PCR assay (Figure 2, Table 4).

Further, it was demonstrated that when one lung cancer cell (A549 cell) was added into 3 ml peripheral blood, *LunX *mRNA could be detectable as a positive case in our established method (data not shown). For NSCLC patients, the positive detection rate of *LunX *mRNA in peripheral blood was high, almost as high as that of *CK19 *mRNA, and much higher than that of *CEA *mRNA (Table 4). Using RT-PCR, KS1/4 was previously reported to be the most sensitive marker among *CEA, CK19, KS1/4, LunX, muc1 *and *PDEF *for the detection of metastatic NSCLC in mediastinal lymph nodes, and *LunX *was with the second highest sensitivity distinguishing lung cancer from lung benign disease [6]. However,*KS1/4 *encodes a glycoprotein that is expressed on epithelial cells and is also present on epithelial cancers, thus, like *CEA *and *CK19, KS1/4 *is not specific to lung tissue. Another novel tumor-specific gene *BJ-TSA-9 *was reported to be a marker for circulating cancer cells in lung cancer patients, but *BJ-TSA-9 *alone was not sensitive enough to detect disseminated cancer cells in peripheral blood, and a combination of *BJ-TSA-9 *with *LunX *and *SCC *was required [7]. *BJ-TSA-9 *also suffers from the same tissue specificity problem that plagues *CEA *and *CK19*. In contrast, expression of the human *LunX *gene is lung-specific, and mRNA could be detected at a concentration of 10^-4 ^μg cancer RNA in 1 μg normal lymph node RNA [1].

A malignant pleural effusion may be the initial presentation of cancer in 10–50% of patients [25]. Cytology is the standard method for the diagnosis of malignant effusion, but the sensitivity of cytology was not good enough [26]. Although an aggressive diagnostic technique thoracoscopy can be used to establish the diagnosis with a higher sensitivity (~90%), this procedure may not be available at all facilities and/or may be too invasive for many patients [27]. The evaluation of tumor markers in pleural fluid thus represents an alternative method for establishing the diagnosis of malignant pleural effusion. In this study, quantitative real-time RT-PCR was performed, for the first time, on pleural fluid. As was the case with peripheral blood, *LunX *mRNA was the most specific marker in malignant pleural fluid, showing a high positive detection rate in lung cancer (13 of 14, 92.9%), compared with the other gene markers *CK19, CEA, VEGF-C *and *hnRNP A2/B1 *mRNA (Figure 4, Table 6). Because determining the presence of circulating cancer cells in the peripheral blood of NSCLC patients is significant for early disease diagnosis and clinical therapy, a test for *LunX *mRNA that is able to reveal small amounts of lung cancer cells in peripheral blood with high specificity and sensitivity would be a great benefit in the clinical management of lung cancer. The potential value of differential *LunX *mRNA expression in pleural fluid for diagnosing malignant effusion reinforces this view.

Recent mass spectroscopy studies indicate that the human *LunX *gene product is also expressed in normal adult nasal lavage fluid. Further, this expression may be up-regulated in response to certain airway irritants, such as cigarette smoke and dimethylbenzylamine [28,29]. Nasal lavage fluid contains a large number of proteins that altogether comprise a potential source for detecting and characterizing biochemical alterations associated with airway diseases. In the present study, nasal lavage fluid samples were not investigated, so the element of smoking was not addressed in comparisons between NSCLC patients, other epithelial cancer patients, benign lung disease patients and healthy volunteers. Furthermore, as shown in Figure 2 and Table 4, *LunX *gene was not detectable in the peripheral blood of healthy volunteers, whether smokers or non-smokers. To date, the functional role of LunX protein remains unknown; however, *LunX *mRNA expression is known to be significantly enhanced in NSCLC tumors compared with corresponding cancer-free lung tissues [1]. In our study, we found that the expression level of *LunX *mRNA in peripheral blood correlated with the pathologic stage of NSCLC (Table 5, Figure 3). The more severe the disease was, the higher the expression level of *LunX *mRNA. Thus, the expression level of *LunX *mRNA in peripheral blood might be a valuable tool for staging NSCLC patients clinically. Furthermore, the expression of *LunX *mRNA in peripheral blood was sensitive to be influenced by the treatment of NSCLC patients (Figure 5), indicating that *LunX *mRNA expression might be associated with lung cancer progression.

## Conclusion

In this study, we demonstrate that the detection of *LunX *mRNA in peripheral blood and pleural fluid by quantitative real-time RT-PCR provides a specific and sensitive indication of the presence of lung cancer cells. To our knowledge, *LunX *mRNA is the most specific NSCLC gene marker currently identified, and has tremendous clinical potential as an NSCLC diagnostic tool. Because *LunX *mRNA levels correlate with clinical severity and change in response to treatment protocols, this marker may prove valuable for NSCLC patients in the clinical decision-making process.

## Competing interests

The authors declare that they have no competing interests.

## Authors' contributions

MC carried out the molecular genetic studies, participated in the sequence alignment and drafted the manuscript. YC performed the statistical analysis and drafted the manuscript. XY conceived of the study and participated in its coordination. ZT participated in the design of the study. HW participated in the design of the study and its coordination. All authors read and approved the final manuscript.

## Pre-publication history

The pre-publication history for this paper can be accessed here:


